# OLDER PEDESTRIANS’ PERSPECTIVES ON WALKING SAFETY IN KAMPALA: A QUALITATIVE INQUIRY INTO THE BUILT ENVIRONMENT

**DOI:** 10.1093/geroni/igae098.3875

**Published:** 2024-12-31

**Authors:** Juliet Namukasa, Heather Mulkey

**Affiliations:** Makerere University Business School, Kampala, Kampala, Uganda; University of Southampton, University of Southampton, England, United Kingdom

## Abstract

Safe, accessible, and inclusive urban environments are important elements in walking as a means of transport. Limited studies are focusing on the walking safety of older people especially in African cities. This study explored the older pedestrians’ perceptions and experiences about walking safety in the built environment of Kampala. A qualitative design was used where purposive and convenience sampling methods were used to recruit fifteen participants from the streets of Kampala and its neighbourhood. Findings showed that older pedestrians had negative perceptions and experiences of walkability and safety in Kampala and its neighbourhoods citing inadequate pedestrian facilities e.g. the lack of sidewalks on most roads, insufficient lighting, uncovered drainage systems, and uneven surfaces. Additionally, the general lack of respect for pedestrians and traffic rules, including speed driving affects the older pedestrians’ sense of safety. Despite these challenges, older pedestrians valued safe mobility within their neighbourhoods for accessing services and sustaining social connections similar to other community members. The study concludes that enhancing the safety of older pedestrians is essential for promoting equitable road access for all members of the community, it urges city policymakers to prioritize improvements in pedestrian infrastructure. Keywords: older pedestrians, walking safety, Kampala City, built environment, perceptions, experiences, qualitative study, equitable road access.

